# Contamination-resistant, rapid emulsion-based isothermal nucleic acid amplification with Mie-scatter inspired light scatter analysis for bacterial identification

**DOI:** 10.1038/s41598-021-99200-4

**Published:** 2021-10-07

**Authors:** Alexander S. Day, Tiffany-Heather Ulep, Elizabeth Budiman, Laurel Dieckhaus, Babak Safavinia, Tyler Hertenstein, Jeong-Yeol Yoon

**Affiliations:** Department of Biomedical Engineering, The University of Arizona, Tucson, AZ 85721 USA

**Keywords:** Biomedical engineering, PCR-based techniques

## Abstract

An emulsion loop-mediated isothermal amplification (eLAMP) platform was developed to reduce the impact that contamination has on assay performance. Ongoing LAMP reactions within the emulsion droplets cause a decrease in interfacial tension, causing a decrease in droplet size, which results in decreased light scatter intensity due to Mie theory. Light scatter intensity was monitored via spectrophotometers and fiber optic cables placed at 30° and 60°. Light scatter intensities collected at 3 min, 30° were able to statistically differentiate 10^3^ and 10^6^ CFU/µL initial *Escherichia coli* O157:H7 concentrations compared to NTC (0 CFU/µL), while the intensity at 60° were able to statistically differentiate 10^6^ CFU/µL initial concentrations and NTC. Control experiments were conducted to validate nucleic acid detection versus bacterial adsorption, finding that the light scatter intensities change is due specifically to ongoing LAMP amplification. After inducing contamination of bulk LAMP reagents, specificity lowered to 0% with conventional LAMP, while the eLAMP platform showed 87.5% specificity. We have demonstrated the use of angle-dependent light scatter intensity as a means of real-time monitoring of an emulsion LAMP platform and fabricated a smartphone-based monitoring system that showed similar trends as spectrophotometer light scatter data, validating the technology for a field deployable platform.

## Introduction

Nucleic acid amplification is a gold standard tool for the identification of target genes-of-interest. Polymerase chain reaction (PCR) is the most used nucleic acid amplification technique which utilizes cyclic temperatures to denature, anneal, and extend in order to create linear copies of the target gene-of-interest in an exponential amount^1^. However, PCR along with other traditional nucleic acid amplification techniques can be limited due to the requirement of pre-processing samples to extract and purify its DNA or RNA.

To monitor in real-time the amplification of PCR reactions, quantitative PCR (qPCR) is also a widely used technology. Fluorescent intercalating dyes that have a high affinity to nucleic acids by embedding between base pairs are utilized and monitored to get exponential curves, such as ethidium bromide and SYBR Green^2^. However, such intercalating dyes can result in non-specific signals because it is non-specific to specific gene-of-interest, but all double stranded DNA (dsDNA). To address this problem, fluorescently tagged hybridization probes that are complementary oligonucleotide sequences to the target gene-of-interest can be utilized^3–5^. Such probes result in fluorescent signals that are specific to the amplification of the target gene sequence rather than non-specific amplification (i.e., primer dimerization). However, creating a novel real-time nucleic acid amplification monitoring system that requires no fluorescent probes or labeling reduces the overall cost and complexity of detection technologies.

Water-in-oil emulsion is often utilized to ensure proper function of nucleic acid amplification platforms and increase the signal-to-volume ratio. This method allows for the compartmentalization of the target-gene-of-interest inside of smaller reaction units. Such technologies can be useful in many research topics that involve amplification steps, as they separate inhibitory components from the reaction, reducing their impact on the overall reaction and reducing cases of non-specific amplification^6,7^. Emulsion platforms have the added advantage due to the necessary prevalence of surfactants and an agitation mechanism during their formation that can be used as a method to “extract” DNA or RNA.

In recent decades, isothermal nucleic amplification methods have been gaining interest because of their ability to utilize a single temperature (when compared to PCR’s temperature cycling nature), thus allowing for the potential mitigation of expensive and specialized laboratory equipment like thermocyclers that require the ability to adjust temperatures finely and rapidly. Such benefits have made these isothermal methods appealing to those who wish to develop field-deployable point-of-care platforms. Such devices would intrinsically need to perform well in the environments where samples might have a higher likelihood of being dirty or contaminated without the need for extensive preprocessing of the sample. However, isothermal nucleic amplification techniques are highly susceptible to non-specific amplification, making them potentially less specific than PCR methods, providing researchers with a hurdle to tackle that could lead to these methods becoming more mainstream^8^. To that end, this study strives to provide a platform that allows for such an increase in detection specificity.

These challenges of mitigating non-specific amplification can be conquered by utilizing alternative methods of measuring nucleic acid amplification. One such method would be to measure the interfacial tension change of the aqueous reaction over time, either directly or indirectly. In our previous study, Harshman et al. used PCR instrumentation that amplified targets-of-interest on a moving droplet-on-a-thermocouple suspended in an oil bath to monitor changes in the droplet size, an indirect measurement of the droplet’s interfacial tension, in real-time. The underlying phenomenon allowing for such detection methodologies is due to amplicon adsorption to the water–oil interface during the ongoing reaction, causing decreases in interfacial tension, which has an impact on many aspects of the aqueous environment^9^. In another study, a droplet loop-mediated-isothermal amplification (LAMP) method monitored interfacial tension by mapping changes in droplet shape in real-time during the LAMP reaction, allowing for real-time detection of pathogens^10^. On both platforms, the overall time-to-result was drastically reduced when compared to conventional nucleic acid amplification methods and allowed for significant amplicon production despite complex sample matrices.

**Figure 1:**
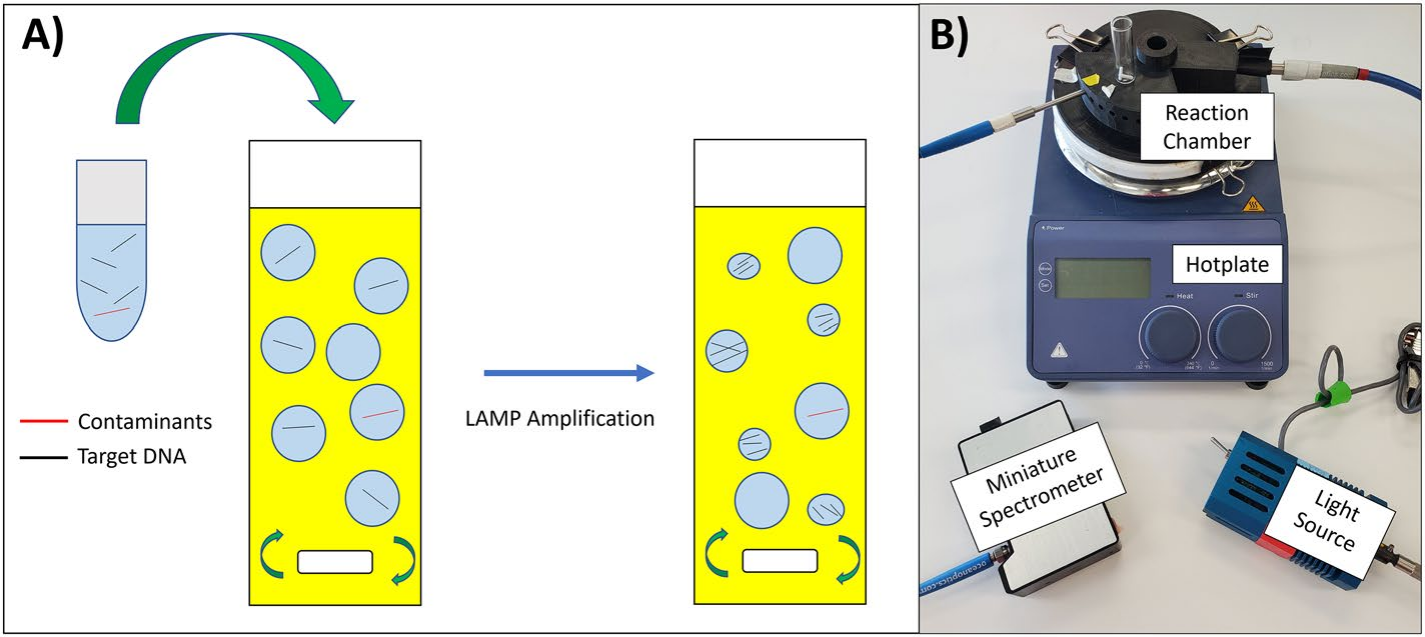
(**A**) Experimental methodology representation shows how potential contaminants are compartmentalized into their nanoliter-sized droplets. As a result, such contaminants cannot adversely affect the amplification (or lack thereof) of the target DNA, thus causing a decrease in droplet size for such amplified droplets. (**B**) A basic schematic illustrating the key materials necessary to conduct eLAMP experiments. It includes a hotplate, 3D-printed hotplate attachment, glass vial with micro stir bar, miniature spectrophotometer, fiber optic cables (to provide the light source and monitor light scatter), and the light source.

This paper focuses on conducting LAMP reactions in a water–oil emulsion platform (emulsion LAMP, or eLAMP) in an attempt to lower the prevalence of non-specific amplification, while also showing reduced reaction times when compared to conventional methods. The mechanism in which we will monitor in real-time the effects of amplification is based on the interfacial changes of micron size emulsions due to amplicon adsorption, followed by emulsion destabilization^11^ (Fig. 1A). These reactions can be monitored via emulsion light scatter, which has its roots in Mie scatter theory, which relates emulsion droplet size to angle-dependent light scatter intensity. This theory is based on previous data collected by our lab^12^, which showed that decreases in droplet diameter (due to amplification of target genes within the droplet) cause a decrease in overall light scatter intensity, for detecting SARS-CoV-2. The detection mechanism was first validated on a platform utilizing a miniature spectrophotometer and fiber optic cables (Fig. 1B), however a smartphone-camera-based platform utilizing blinking red LEDs for light sources was also investigated. A decrease in light scatter intensity at multiple angles was observed in relation to initial target concentration within just 3 min of the emulsion LAMP reaction for both detection set-ups. Overall, we have demonstrated the use of emulsion-guided angle-dependent light scatter intensity measurements as a means of monitoring isothermal nucleic acid amplification reactions in real-time using a simple and user-friendly platform that shows increases in assay specificity when compared to conventional platforms.

Compared to earlier emulsion LAMP works by our lab^12^, this project aims to amplify and detect bacteria samples that are relatively “dirty.” *Escherichia coli* O157:H7 was used as a model bacterial target, amplifying rfbE gene. While the same bacteria species was also used in^12^, it was used only for preliminary optimization and amplified only in a conventional manner. We also investigate the role of protein adsorption to the water–oil interface towards altering the interfacial tension. We aim to show that the overall sensitivity and specificity of the platform is superior to conventional amplification methods.

## Results

### DNA fragment mixture as model LAMP amplicon products in emulsion platform

**Figure 2:**
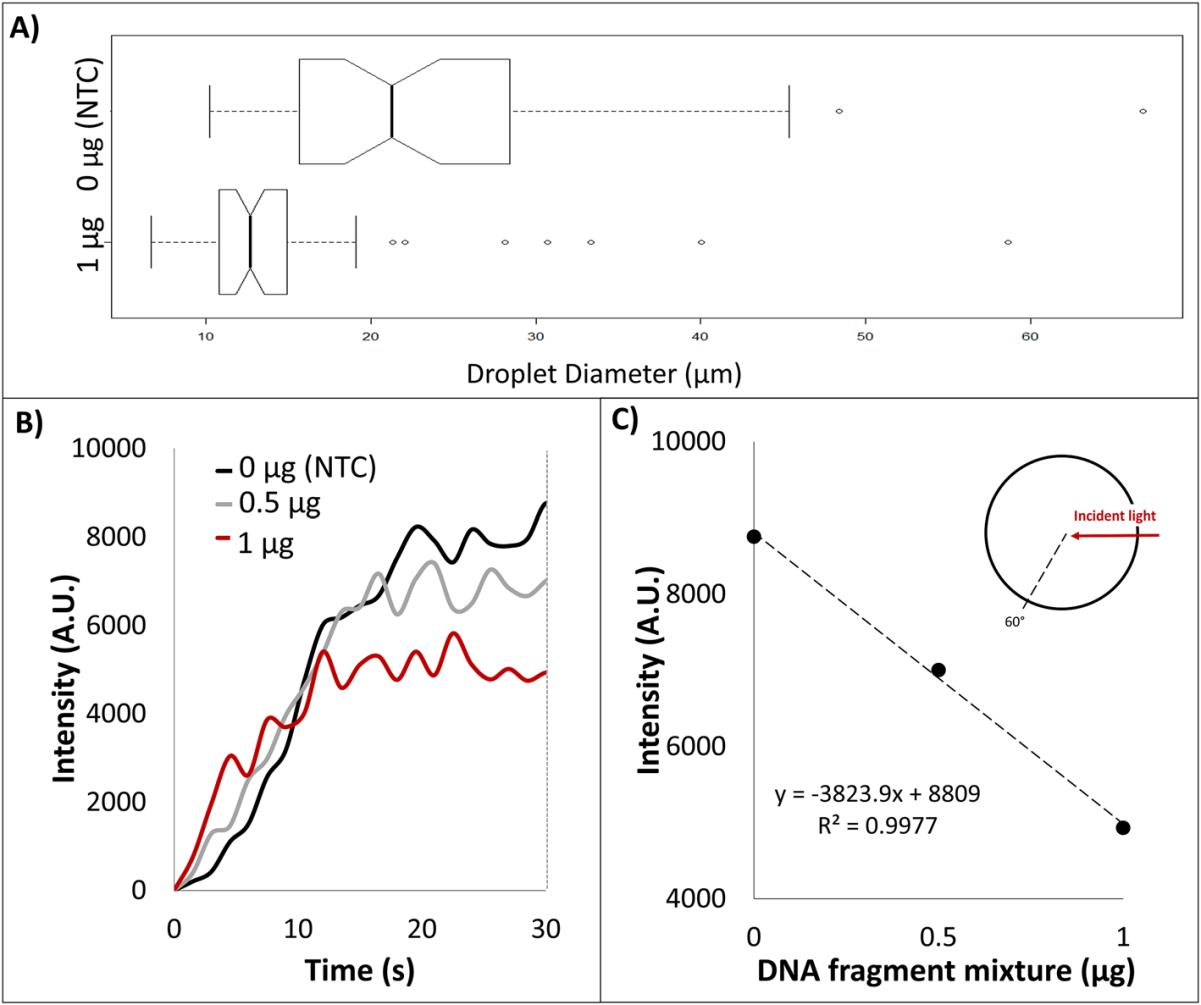
(**A**) Measured diameter from light microscope images of water–oil emulsions containing 10 µL of aqueous 1 and 0 µg DNA fragment mixture. (**B**) Emulsion light scatter intensity at 60° with respect to 650 nm incident light over time of 1, 0.5, and 0 µg DNA fragment mixture in 10 µL aqueous phase. (**C**) The 60° intensities at 30 s plotted against the DNA fragment amount.

As a model sample matrix to simulate LAMP amplicon production, a DNA fragment mixture was again utilized to determine diameter size and light scatter intensity in relation to concentration of DNA present in the emulsion platform. Figure 2A shows the diameter distribution between an emulsion sample with and without DNA fragment mixture in the emulsion platform after 1 min of agitation. The average diameter size for emulsion with and without DNA was 23.4 pixels and 15.0 pixels, resulting in a 35.8% difference. Therefore, due to the presence of DNA, emulsion diameter size is decreased due to decreased interfacial tension at the oil water interface rendering unstable emulsions, resulting in smaller diameters.

As demonstrated previously, light scatter, supported by the Mie theory, will change in intensity due to size dependencies. In Fig. 2B, light scatter intensity at 60° for 1, 0.5, and 0 µg DNA fragment mixture is collected in relation to time. Within the first 15 s, light scatter intensity is independent of concentration of DNA fragment mixture. However, light scatter intensity at 30 s in relation to concentration of DNA fragment mixture shows a linear relationship (R^2^ = 0.997) (Fig. 2C). The percentage change between no DNA fragment mixture (nuclease-free water) and 1 µg was a 43.6% difference. It can then be alluded, that the decrease in intensity is due to an increase amount of amplicon product due to a decrease distribution of emulsion diameter size.

This data, combined with our lab’s previously published results (showing that increasing amounts of conventionally amplified LAMP amplicons cause a decrease in reaction interfacial tension and a subsequent decrease in light scatter intensity of emulsified reactions), provide evidence to support the hypothesis that ongoing LAMP amplification within the emulsified reaction should lead to a decrease in light scatter intensity^12^.

### Angle-dependent light scatter collection via miniature spectrophotometer

**Figure 3:**
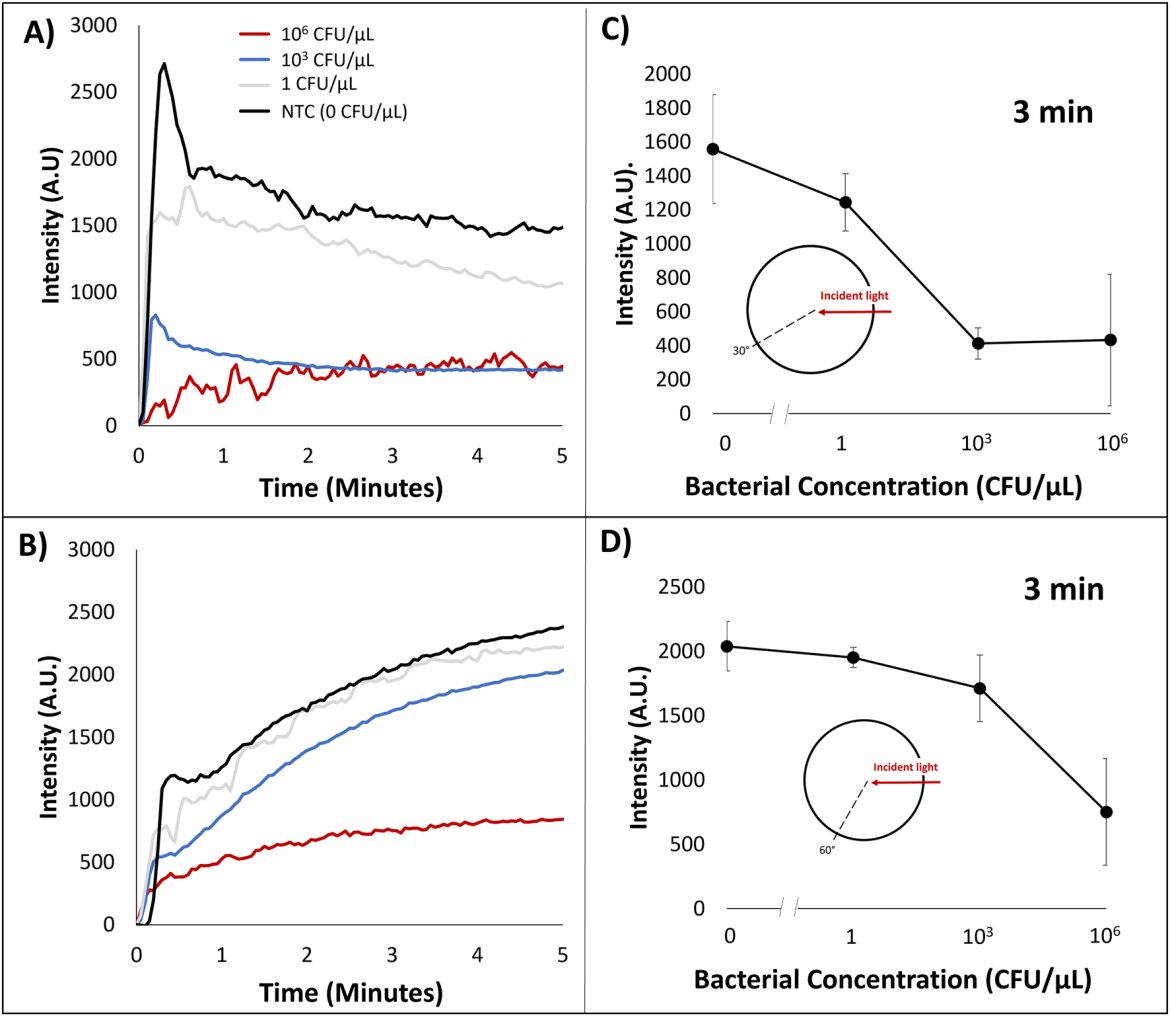
Emulsion LAMP light scatter intensity via spectrophotometer over time at (**A**) 30° and (**B**) 60° angle with respect to 650 nm incident wavelength with varying initial bacteria concentration of 10^6^, 10^3^, 1, and 0 CFU/µL. (**A** and **B**) are the representatives chosen from 3 replicates for each concentration. Light scatter intensity at 3 min for (**C**) 30° and (**D**) 60° angle for bacteria concentrations of 10^6^, 10^3^, 1, and 0 CFU/µL.

Light scatter intensity from fiber optic cables placed at 30° and 60° angles with respect to a 650 nm incident light was collected from a miniature spectrophotometer of emulsion samples with LAMP reactions containing initial bacteria concentrations of 10^6^, 10^3^, 1, and 0 CFU/µL. Representative raw intensities plotted over time are shown in Fig. 3A, B, while the average intensities (3 replicates for each concentration) at 3 min are shown in Fig. 3C, D. Significant differences (*p* < 0.05) could be observed after 3 min. Before that time, the initial spikes in light scatter intensities significantly varied from sample to sample, hence no significant differences, which gradually converged to the average values after 3 min. For both angles the underlying trend was that light scatter intensity decreased with increasing concentration (Fig. 3C, D). This trend is synonymous to trends found with light scatter of emulsions containing varying concentration of DNA fragment mixture. Light scatter intensities collected at the 30° angle showed the greatest difference in change in comparison to NTC (0 CFU/µL) for 10^6^ and 10^3^ CFU/µL. The greatest percent change in light scatter intensity appeared to be 69.7% within 3 min on the emulsion platform between 0 and 10^3^ CFU/µL. 60° light scatter intensities at different initial bacteria concentration did not show significant differences other than 10^6^ CFU/µL. Light scatter intensity at 3 min shows a 20.6% intensity fluctuation amongst 0 to 10^3^ CFU/µL concentrations, followed by a significantly different 79.3% intensity change in comparison to NTC for 10^6^ CFU/µL (Fig. 3C, D).

### Control experiments with no LAMP reagents

**Figure 4:**
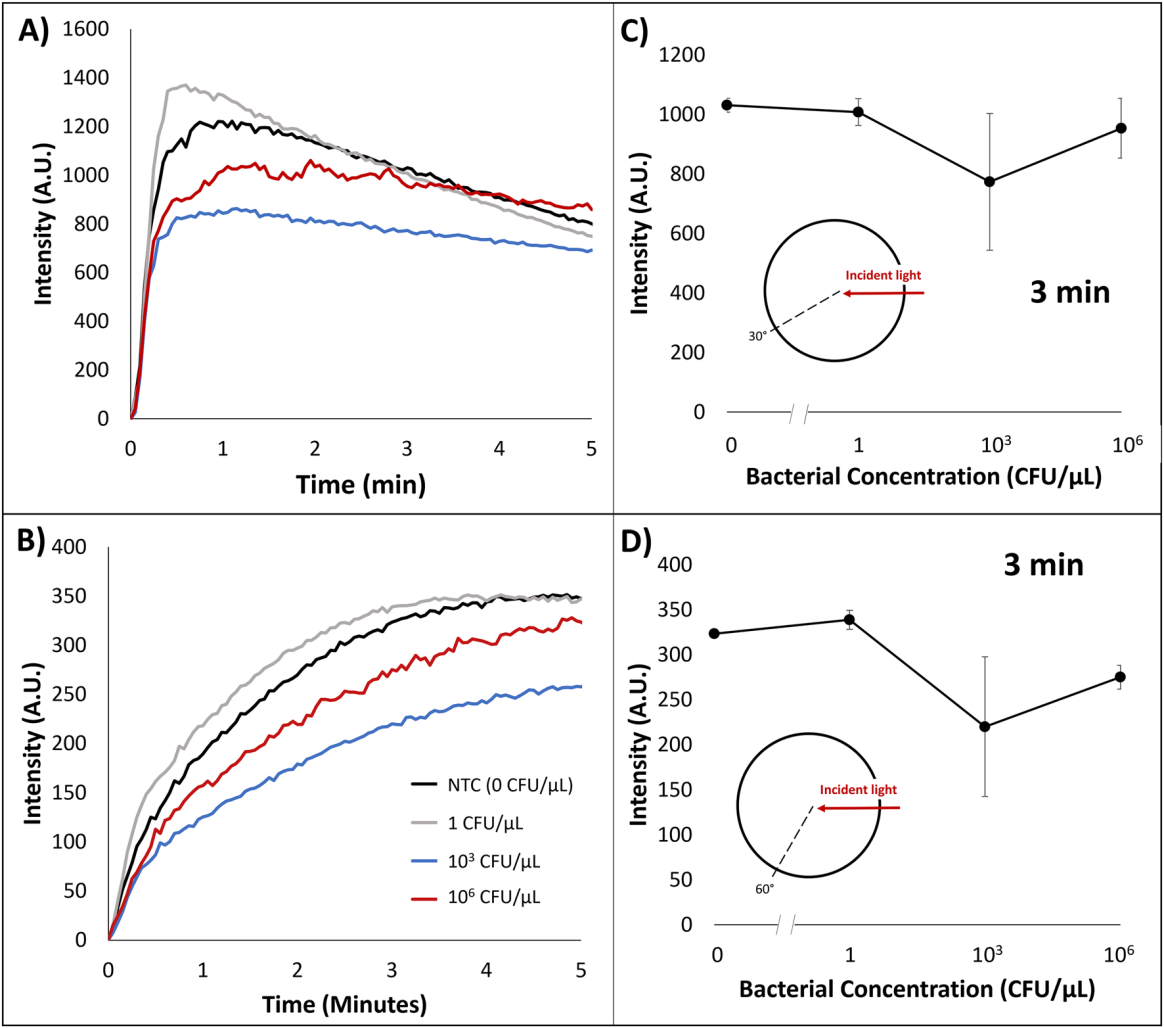
Emulsion LAMP light scatter intensity with relation to time collected at (**A**) 30° and (**B**) 60° angles with respect to a 650 nm wavelength incident light with bacteria solution droplets (along with BSA as stabilizer), varying concentrations of 10^6^,10^3^, 1, and 0 CFU/µL. (**A** and **B**) are the representatives chosen from 3 replicates for each concentration. Light scatter intensity at 3 min collected from (**C**) 30° and (**D**) 60° angles at various bacterial concentrations.

Control experiments were conducted on the emulsion platform with the addition of 10 µL aqueous solutions containing diluted bacteria concentrations of 10^6^,10^3^, 1, and 0 CFU/µL with bovine serum albumin (BSA) as a stabilizer. Bacteria solutions with no LAMP reagents were used to investigate whether the light scatter changes were originated indeed from nucleic acid amplification or initial adsorption of bacteria cells and fragments. Representative raw intensities plotted over time are shown in Fig. 4A, B, while the average intensities (3 replicates for each concentration) at 3 min are shown in Fig. 4C, D. Light scatter intensities were collected at 30° over time for three different bacteria concentrations, and average intensities are summarized in Fig. 4C. Overall, there is no statistically significant concentration dependency (utilizing Pearson’s T-test), indicating that 30° light scatter intensities were not affected by initial bacterial concentrations and this detection method can be used to determine nucleic acid amplification dependencies.

Utilizing the same emulsion samples, 60° light scatter intensities were also collected over time (Fig. 4B) and plotted in relation to bacteria concentration at 3 min (Fig. 4D). Results are similar to those with 30° light scatter intensities. Therefore, we can conclude that the light scatter intensities can be used to determine target nucleic acid presence and its subsequent amplification via emulsion LAMP.

### Angle-dependent light scatter collection via smartphone camera

**Figure 5:**
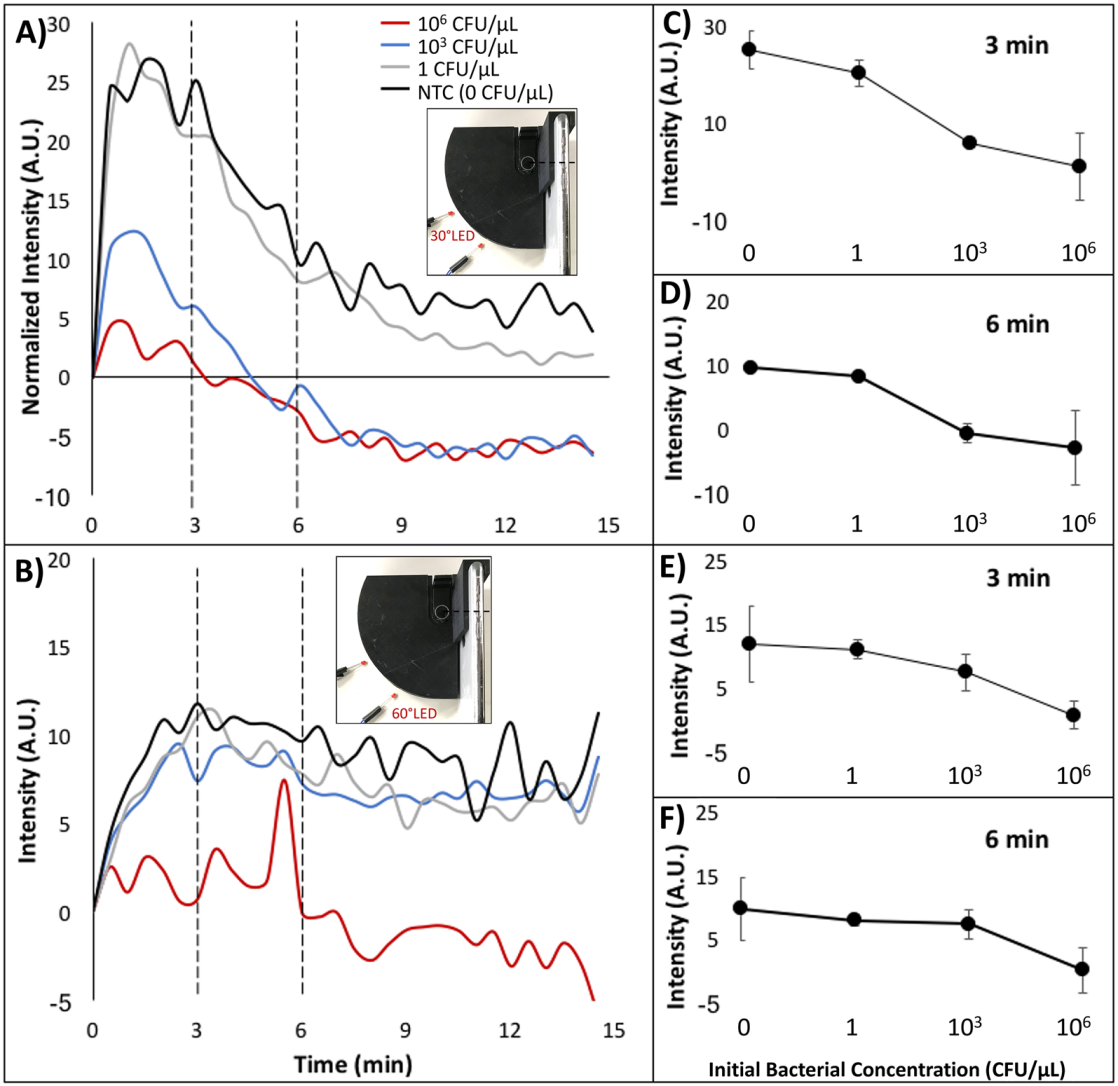
Emulsion LAMP light scatter intensity via smartphone camera over time at (**A**) 30° and (**B**) 60° angle with respect to 650 nm incident wavelength with varying initial bacteria concentration of 10^6^, 10^3^, 1, and 0 CFU/µL. A and B are the representatives chosen from 3 replicates for each concentration. 30° light scatter red channel intensity at (**C**) 3 min and (**D**) 6 min. 60° light scatter red channel intensity at (**E**) 3 min and (**F**) 6 min with bacteria concentrations of 10^6^, 10^3^, 1, and 0 CFU/µL.

A 3D printed hot plate attachment was designed and fabricated to hold the emulsion reaction chamber and house two blinking red LEDs placed at 30° and 60° angles with respect to a smartphone camera. 10 µL LAMP reactions with varying initial bacteria concentrations of 10^6^, 10^3^, 1, and 0 CFU/µL were placed into the emulsion platform in a similar fashion as the spectrophotometer procedure. BSA was not added. Images were taken every 3 s synced to the differently angled LEDs over the course of 15 min to characterize smartphone optical detection as replacement for a spectrophotometer and fiber optical cable experimental set up for a more user-friendly platform. From the captured images, red channel intensity was extracted and sorted from time-lapsed image sequence for both angles (Fig. 5A and B). Intensity from these curves were taken in relation to concentration of initial target concentration at 3 min and 6 min. Similar trends were found for both angles, as initial target concentration increased, intensity decreased. 30° light scatter intensity at 3 min and 6 min showed a 96.3% and 131% change in intensity between NTC and 10^6^ CFU/µL (Fig. 5C and D). 60° light scatter intensity at 3 min and 6 min showed a 93.8% and 102% change in intensity between NTC and 10^6^ CFU/µL (Fig. 5E and F).

Interestingly, the percent changes in intensities in comparison to NTC across bacteria concentration collected via smartphone camera were larger in comparison to intensities collected via spectrophotometer. This could be due the ability to resolve outlier reactions based on image observations. For example, a reaction was omitted from the data set when a large bubble was accidentally introduced into the emulsion. Such omission could not be conducted on a spectrophotometer set up because there are no images to confirm such event. However, disadvantages to utilizing a smartphone to capture light scatter intensities via time-lapse is it is not conducted in a real-time fashion. Images must be taken from the smartphone, uploaded, and processed via Python automation script. Next steps to address this concern is rather than a smartphone as the optical transducer, a microcontroller camera connected to a smartphone user interface could be designed as a standalone device.

### Sensitivity and specificity of contaminated samples

**Figure 6:**
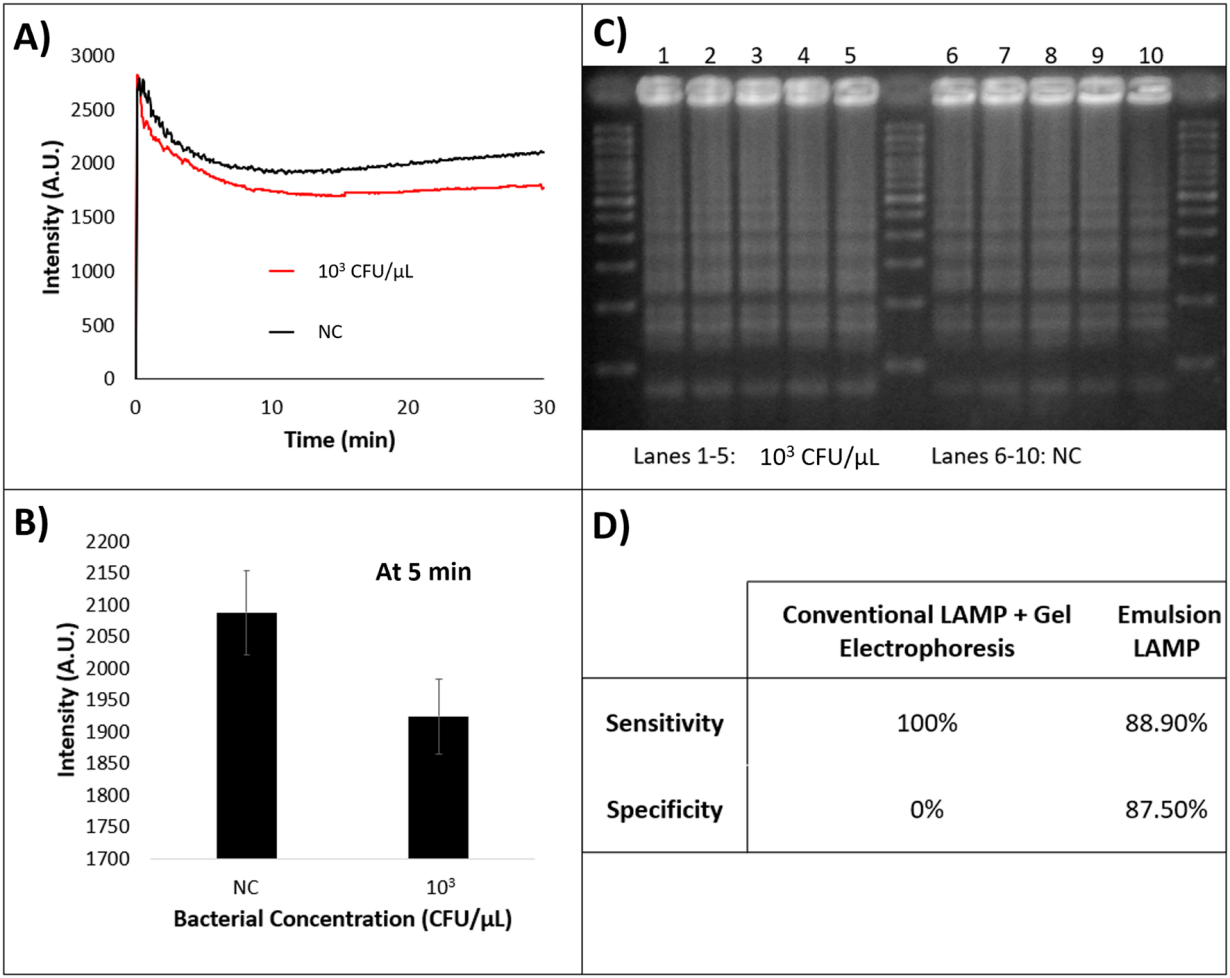
Sensitivity and specificity in comparison to conventional LAMP. (**A**) Average 30° light scatter intensity profiles for 10^3^ CFU/μL and NC (negative control) samples. (**B**) Comparison of light scatter intensity values at 5 min between initial target bacterial concentrations. (**C**) Gel image showing amplification of both positive (left lanes) and NC (right lanes) samples after conventional amplification for 30 min. (**D**) Table detailing sensitivity and specificity of both conventional amplifications combined with gel electrophoresis and emulsion LAMP.

The emulsion platform was further tested for the bacterial samples prepared in a contaminated (identified as *Salmonella* Typhimurium, which contains the genes similar to *E. coli*) biosafety cabinet. These results were compared with conventional amplification combined with gel electrophoresis, and presence of amplification on the gel was used to determine conventional sensitivity and specificity. The sensitivity and specificity of the emulsion platform were measured by running 9 positive control (PC) samples (with a target bacterial concentration of 10^3^ CFU/μL) and 8 negative control samples (NC samples) (Fig. 6A and B), then utilizing the interquartile range outlier detection method to determine if any sample’s light scattering intensity value at 5 min tended to behave more like samples belonging to the other test group. Using this design, we found that 1 of the 9 PC samples and 1 of the 8 NC samples behaved as an outlier, indicating that the assay has an apparent sensitivity of 88.90% and a sensitivity of 87.5% (Fig. 6D). This was compared to the sensitivity and specificity of conventionally amplified (LAMP) samples from bacterial samples ran through gel electrophoresis, which was found to be 100% and 0%, respectively (Fig. 6C and D). It is believed that the presence of a non-target bacteria in the NC samples (from the underlying contamination of the biosafety hood) and the use of whole bacteria targets are what caused all NC samples to amplify on the conventional platform due to the widespread availability of non-target sequences and primer mixes, which is not apparent in each individual reaction volume on the emulsion LAMP platform.

**Figure 7:**
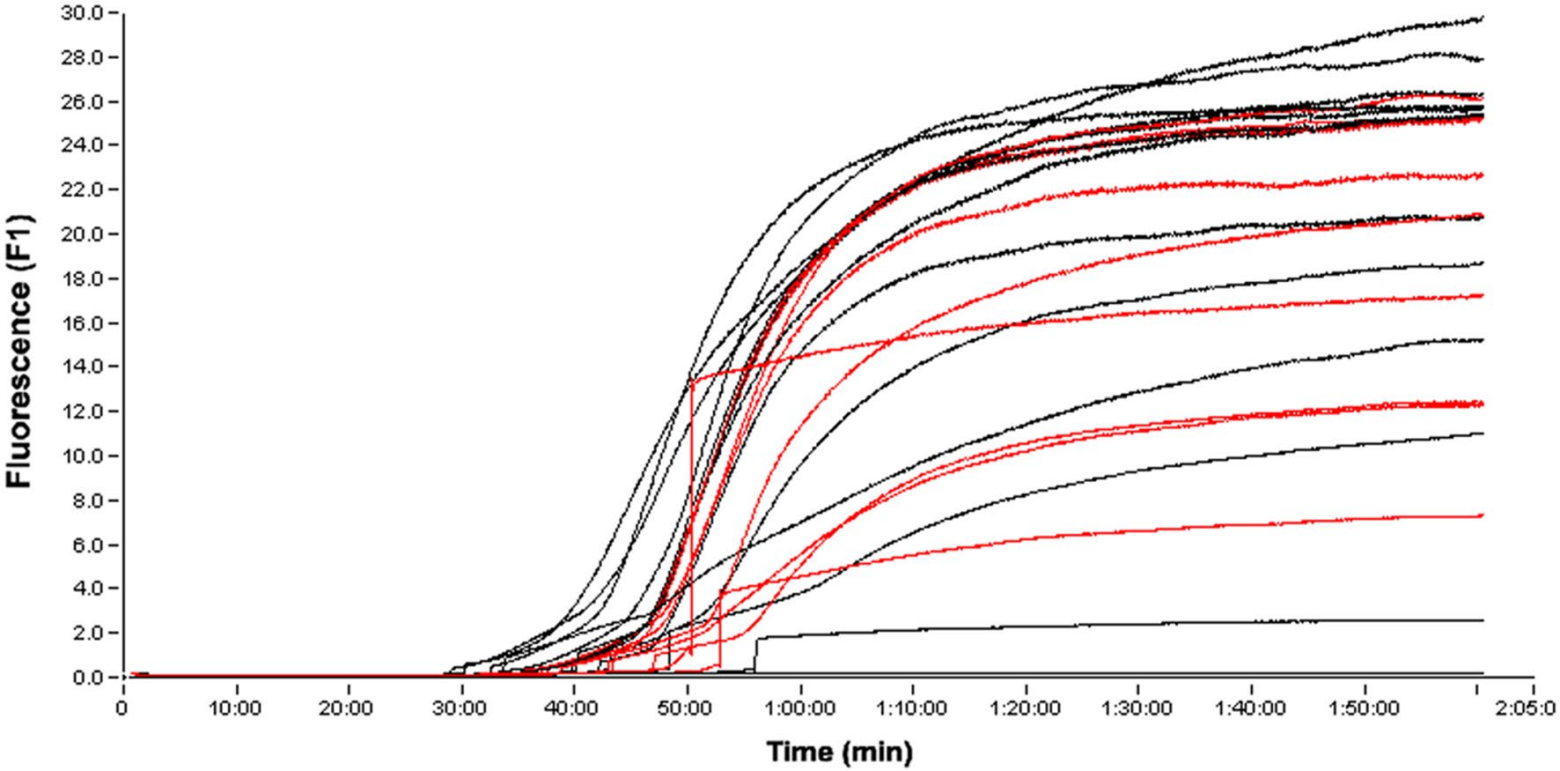
Measured fluorescence of conventional LAMP amplification of positive control samples (red lines, n = 13) and negative control samples modeling contamination (black lines, n = 12).

This comparison with gel electrophoresis is compounded with the results acquired from running multiple positive and negative control samples on a Roche LIGHTCYCLER system, as shown in Fig. 7. These results show no discernable distinction between amplification times for the two sample types, indicating the lower specificity of conventional LAMP platforms. A separate agarose gel can be seen in Supplementary Fig. S1, where the gel indicates that the amplification present in both the contaminated samples (negative controls) and target samples (positive controls) was not present in no target controls (NTCs), thus indicating that the amplification seen on all three platforms (emulsion LAMP, conventional amplification via thermocycler, and fluorescent amplification via Roche LIGHTCYCLER) was not simply that of eventual nonspecific amplification seen in amplification reactions that go on for extended time periods. Regardless, the sensitivity of the emulsion platform was slightly lower than that of the gold standard method, but the specificity was significantly higher as well when compared to both conventional platforms.

## Discussion

In this study, an emulsion-based LAMP platform was utilized to investigate whether monitoring angle-dependent light scatter in real-time could both reduce the time-to result for nucleic acid amplification technologies, as well as decrease the prevalence of non-specific amplification when compared to conventional isothermal amplification due to the compartmentalization of the LAMP reaction. The underlying phenomenon attributing changes in reaction interfacial tension (IFT) due to ongoing nucleic acid amplification was first verified via pendant droplet analysis of samples containing varying amounts of DNA fragment mixture as a substitute for amplicons. The measurements showed that added DNA fragment mixture caused a decrease in IFT. Therefore, increasing DNA presence at the water–oil interface destabilizes the droplet, causing changes in droplet IFT. Such changes in IFT would then cause a decrease in droplet diameter on the emulsion platform, which was verified via light microscope images of samples containing DNA fragment mixture. For example, samples containing no DNA fragments (NTC, or 0 µg DNA fragment mixture) had an average emulsion diameter 43.6% smaller than samples containing model amplicons (1 µg DNA fragment mixture). In addition, adding varying amounts of model amplicons showed a linear decrease in 60° angle-dependent light scatter intensity, providing confirmation that the changes in droplet diameter resulted in representative changes in light scatter due to Mie scatter^13^.

LAMP emulsions with varying initial bacteria concentrations were performed while light scatter intensity at 30° and 60° were monitored in real time. Intensity light scatter values at 3 min showed similar trends to the light scatter experiments with DNA fragment mixture solutions. At 3 min, 30° light scatter intensity can statistically differentiate 10^3^ and 10^6^ CFU/µL initial concentrations in comparison to NTC (0 CFU/µL). 3 min light scatter intensities collected at 60° can statistically differentiate 10^6^ CFU/µL initial concentrations in comparison to NTC (0 CFU/µL).

As shown in Figs. 3 through 5, significant differences in light scatter values could be observed as early as 1 min, although no statistical difference could be observed (*p* > 0.05). Initial spikes varied significantly from sample to sample, which converged to the average values after 3 min. The number of LAMP amplicons would be small for the first 1 min, while they may be nonetheless sufficient to alter the interfacial tension at the water–oil-interface. Harshman et al. have previously demonstrated a detectable change in interfacial tension during PCR with a 5–10 µL droplet^9^. With LAMP and much smaller emulsion droplets (10 µL diameter = 0.5 pL)^12^, this could be reduced to 3 min reproducibly and 1 min occasionally. Since the doubling time of typical LAMP has been reported as fast as 30 s^5^, 2 doublings (4 times) could be possible after 1 min and 12 doublings (4096 times) after 3 min. As we added the LAMP reaction mixture dropwise to the pre-heated oil, some molecules could have been pre-exposed to heat before the 0-min mark.

**Figure 8:**
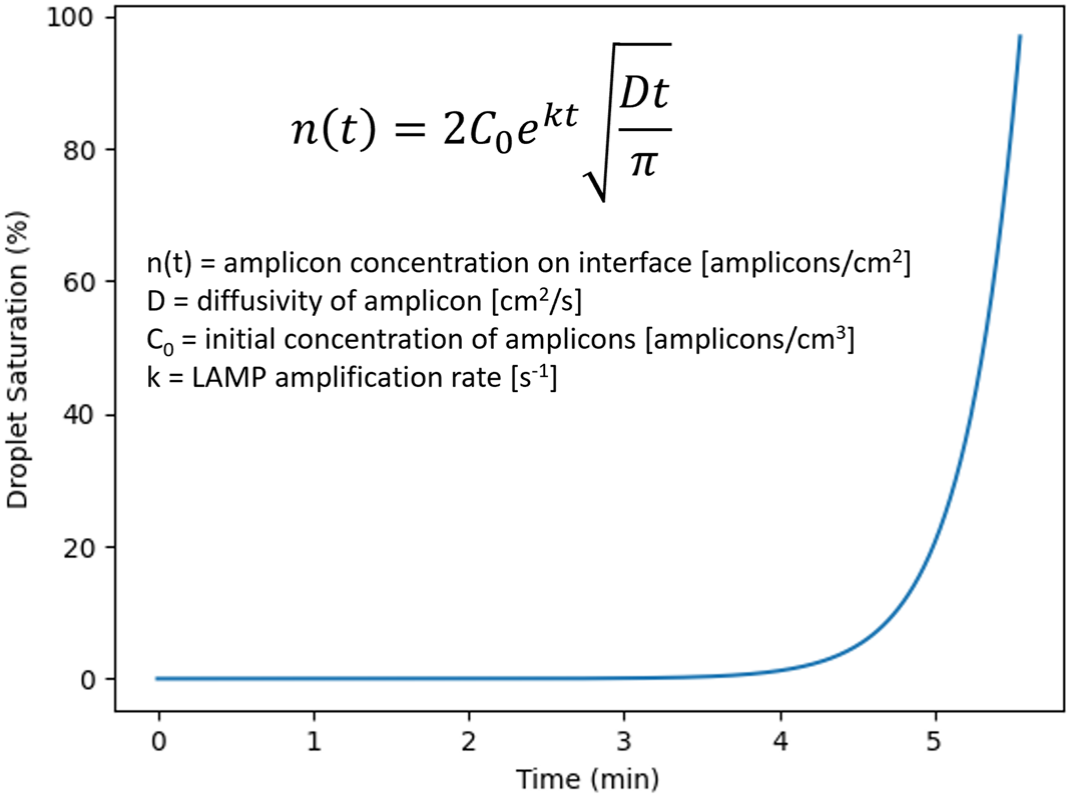
Theoretical model equation and modeling results of LAMP amplicon creation and subsequent adsorption to the oil–water interface within the emulsified droplets.

We utilized a theoretical mathematical model combining the Fick’s diffusion equation with an exponential growth model of LAMP amplicon creation to model the oil–water interface saturation with LAMP amplicons over time^14^. This model provides a theoretical framework modeling how fast the emulsion droplet should change its interfacial tension due to amplicon adsorption, thus providing a model for how quickly significant light scatter differences can be seen on the platform. The results of this model and the equation used can be found in Fig. 8, which indicates that complete saturation of the emulsified droplet’s surface area with LAMP amplicons happens in ~ 5 min. Keeping in mind that this model does not account for initial adsorption of other reaction enzymes or bulk proteins (such as the BSA present for droplet stabilization), this model indicates that sub-ten-minute detection times are theoretically possible.

As a control study to determine if light scatter changes were due to initial bacterial adsorption as opposed to amplicon adsorption, solutions of varying bacteria concentrations with no LAMP reagents were placed into an emulsion and monitored for light scatter. At both detection angles, there was no significant concentration dependency, indicating that the changes in light scatter intensity were not due to bacterial adsorption, but instead due to amplicon adsorption as the LAMP reaction occurred.

A 3D printed hot plate attachment was utilized to allow for smartphone monitoring of the reaction in conjunction with two blinking red LEDs placed at 30° and 60° relative to the smartphone camera to simplify the emulsion platform. The results pertaining to this platform showed similar light scatter intensity decreases with increasing initial bacterial concentration, thus demonstrating how this platform could be translated into a field-deployable device to be used in resource-limited settings as well as the clinical space.

Finally, the emulsion LAMP platform was utilized specifically in a setting where contamination had been proven to affect conventional LAMP reactions, and the emulsion platform showed an overall decrease in non-specific amplification (0% specificity with conventional LAMP and 87.5% specificity with emulsion LAMP, both using contaminated samples), thus rendering it potentially more specific than conventional LAMP platforms.

## Materials and methods

### Preparation of specimen

Stock *Escherichia coli* O157:H7 (part #0801622; ZeptoMetrix, Buffalo, NY, USA) bacterial solution was diluted to concentrations of 10^6^, 10^3^, 1, 0.1, and 0 CFU/µL in nuclease free water. DNA fragment mixture (10488058; ThermoFisher Scientific, Waltham, MA, USA) was diluted in nuclease-free water to different amounts of 1, 0.5, 0.1, and 0 µg as a model sample for final LAMP amplicon products.

### Light scatter detection

A red incident light at 650 nm (LS-450 LED; Ocean Insight, Orlando, FL, USA) allowed for the illumination of the emulsion samples via a fiber optical cable, while 2 fiber optical cables connected to a miniature spectrophotometer (USB4000, Ocean Insight) placed at 30° and 60° collected light intensity. Light scatter intensity measurements were collected every 3 s for both the smartphone and spectrophotometer platforms. A 3D printed attachment previously utilized by our lab^12^ was used to secure the optical instruments and vial containing the emulsion reaction throughout the assay. Experiments were duplicated, replacing the miniature spectrophotometer with a smartphone camera (iPhone 9). Images were collected using the time lapse feature, capturing images every second. Exposure and white balance were fixed. Red channel was isolated and cropped to represent the emulsion suspension that is excited. Average red intensity was collected from each image.

### Emulsion DNA fragment light scatter detection and diameter measurement

10 µL DNA fragment mixtures of 1, 0.5, and 0 µg were placed into pre-heated 65 °C oil phase with a 650 nm incident light illuminating on sample. At the 60° angle, bulk light scatter was collected via fiber optic cable and spectrophotometer. 10 µL of the emulsion was also collected at 1 min, when sufficient emulsion formation was succeeded. Microscope images were then post-processed and measured in ImageJ software (US National Institutes of Health; Bethesda, MD, USA).

### LAMP reaction

LAMP primers were found from literature^15^ and their oligonucleotide sequences can be found in Supplementary Table S1. They were purchased from Sigma-Aldrich (St. Louis, MO, USA). 10× primer sets were created to contain 16 µM each of FIP and BIP primers, 8 µM each of Loop-F and Loop-B primers, and 2 µM of F3 and B3 primers. LAMP reactions were prepared on ice and utilized the WARMSTART LAMP Kit DNA & RNA (E1700; New England Biolabs Inc, Ipswich, MA, USA). The final LAMP mixture contained 5:1:0.4:1:2.6 ratio of Warm Start LAMP 2× master mix, 10× primer mix, target bacteria dilution (or nuclease-free water for no target control, NTC), 20 mg/mL bovine serum albumin (B8667; Sigma), and nuclease-free water. Conventionally amplified samples were conducted in a thermocycler (MJ Research, Waltham, MA, USA) programmed to run at 65 °C for 30 min, followed by a refrigeration step occurring at 4 °C.

### Emulsion LAMP assay

Water-in-oil colloidal emulsions were prepared in the same fashion as our previously published method^10^. An emulsion reaction consisted of 2 mL of preheated (65 °C) oil phase followed by dropping a suspended 10 µL aqueous LAMP droplet from a blunt end needle. Emulsions were formed and agitated by a micro stir bar set to 1500 rotations per minute (RPM) for the entirety of the reaction. Experimental setup is shown in Fig. 1B. Post-reaction, emulsions were collected and the end byproducts were extracted using 3 iterations of organic purification with water saturated diethyl ether^16^.

### End-point amplification analysis

After emulsions assay was performed then broken, the amplicon precipitate dissolved in an aqueous solution was analyzed. Presence of nucleic acid was determined by measurement of absorption at 260 nm. LAMP products were analyzed using gel electrophoresis. 3% w/v agarose gel (A0169; Sigma-Aldrich) in 1X tris–acetate-EDTA (TAE) buffer (35100131; Quality Biological Inc, Gaithersburg, MD, USA) was prepared and placed at 120 V for 50 min with an electrophoresis power supply (FB200; ThermoFisher Scientific). TRACKIT 100 bp DNA ladder was used as a standard for fragment sizing. Gels were stained with ethidium bromide (E1510; Sigma-Aldrich) and imaged under UV light. Gel images were analyzed using ImageJ software (US National Institutes of Health).

### Preparation of contaminated samples

After observing widespread laboratory contamination originating from a contaminated biosafety cabinet, a fresh master mix bulk solution was aliquoted within the said biosafety cabinet to mimic how common laboratory contaminations can occur. Such contamination was validated using conventional amplification combined with gel electrophoresis, and presence of amplification on the gel was used to determine conventional sensitivity and specificity. 8 samples of NC (no target was added deliberately, however contaminated master mix aliquots were used) and 9 samples of 10^3^ CFU/µL (positive control) were then ran on the eLAMP platform, and the light scatter intensities were compared by running an outlier test on each sample set. If an outlier was seen in the NC samples, the conclusion was made that it amplified, whereas if an outlier was seen in the 10^3^ CFU/µL, the conclusion was that it did not amplify. These datapoints were then used to calculate assay sensitivity and specificity.

### Fluorescent LAMP specificity experiments

To further model the increased specificity, we ran fluorescent LAMP experiments on a Roche LIGHTCYCLER programmed to run at 65 °C for 2 h using the same reaction mixture used previously in the study, but with an added 1 µL of New England dye. 13 samples containing 10^3^ CFU/µL target bacteria (positive control) and 12 samples containing 10^3^ CFU/µL *Salmonella* Typhimurium Z005 strain (ZeptoMetrix, NY, USA) as a contaminating bacterium (negative control) were run on the LIGHTCYCLER to test a conventional LAMP platform’s specificity for the target bacterium. The results are compared with the gel electrophoresis results seen in the previous section to evaluate the overall specificity of both these conventional LAMP platforms.

### Theoretical modeling of LAMP amplicon creation and adsorption

To mathematically model amplicon creation during the ongoing LAMP reaction and the subsequent adsorption of the amplicons to the oil–water interface, we utilized an equation similar to the one used in our previously published study^10^. The final equation can be seen in Fig. 8, which is a combination of Fick’s diffusion equation with an exponentially increasing concentration of amplicons (due to the LAMP reaction). We used this equation to provide a theoretical standard for how fast the emulsion droplets saturate with amplicons. The standard emulsion droplets size used in the model was the median measured droplet size using microscopic imaging in our previously published study. The main assumptions of the model were that the modeled droplet started with a single target gene copy, and the rate of LAMP reaction and amplicons diffusivity are calculated from the literature^10,12^. The model was run using Python, and the model code can be found hosted on GitHub (yoon-bsl/eLAMP-Modeling).

## Supplementary Information

Supplementary Information 1.

Supplementary Information 2.

## Data Availability

All data generated or analyzed during this study are available in the Supplementary File.
