# Supplementary material for: Contamination-resistant, rapid emulsion-based isothermal nucleic acid amplification with Mie-scatter inspired light scatter analysis for bacterial identification
Source: Sci Rep. 2021 Oct 7;11:19933. doi: 10.1038/s41598-021-99200-4 (PMC8497611; doi:10.1038/s41598-021-99200-4)
Supplement: Supplementary file 2 — Supplementary Information 2. [file 41598_2021_99200_MOESM2_ESM.pdf]

Supplementary Information

Contamination-resistant, rapid emulsion-based isothermal nucleic acid amplification with Mie-scatter inspired light scatter analysis for bacterial identification

Alexander S. Day†, Tiffany-Heather Ulep†, Elizabeth Budiman, Laurel Dieckhaus, Babak Safavinia, Tyler Hertenstein, and Jeong-Yeol Yoon*

Department of Biomedical Engineering, The University of Arizona, Tucson, Arizona 85721, United States

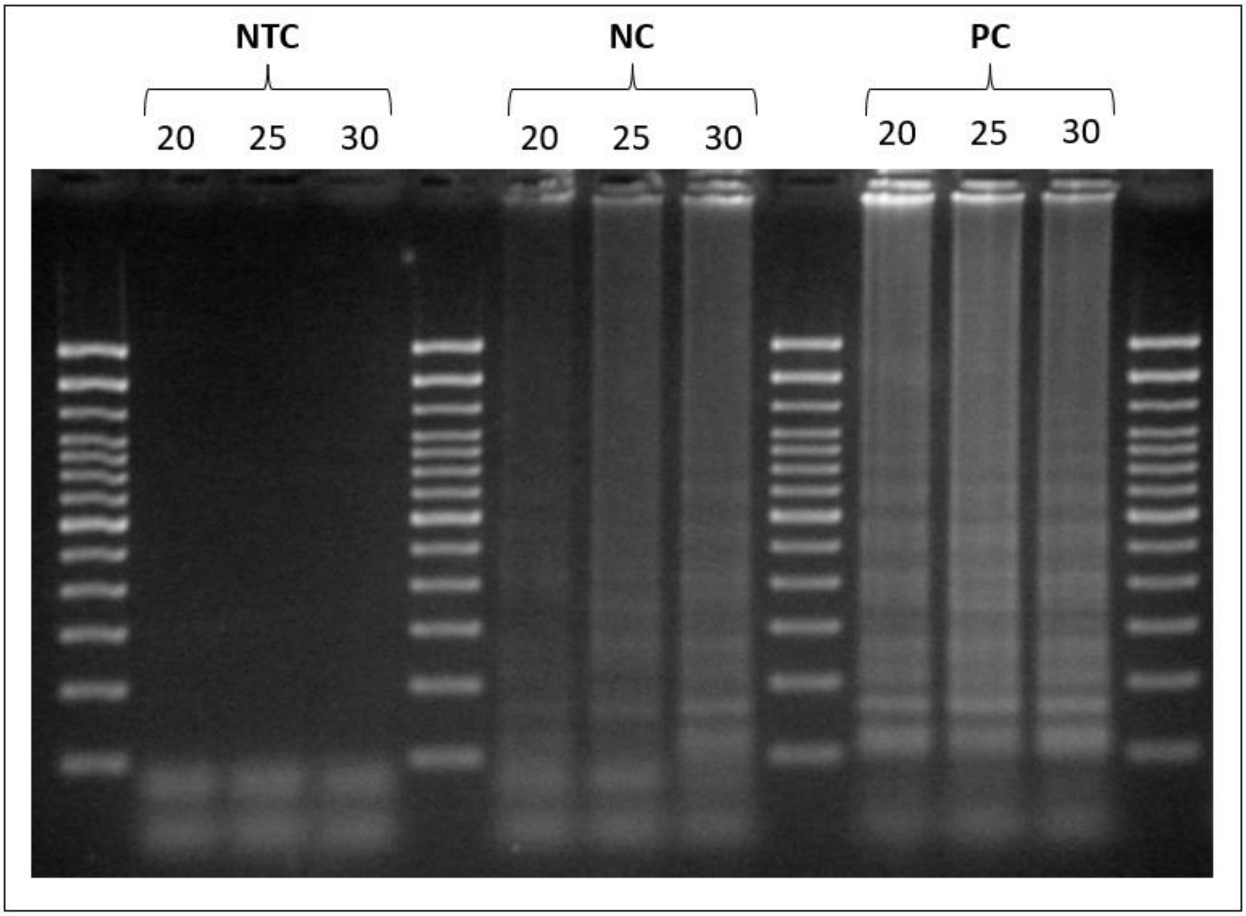

Supplementary Figure S1. Gel images indicating lack of no target control (NTC) LAMP amplification from 20 to 30 minutes and prevalence of LAMP amplification from 20 to 30 minutes for both negative control (NC) and positive control (PC) samples

Supplementary Table S1. Oligonucleotide LAMP primer sequences

Target	LAMP Primer	Sequence (5'-3')	Size (bp)
E. coli O157:H7 *rfbE* gene	F3	AACAGTCTTGTACAAGTCCA	20
	B3	GGTGCTTTTGATATTTTTCCG	21
	FIP	CTCTCTTTCCTCTGCGGTCCGATGTTTTCACACTTATTGGAT	43
	BIP	TAAGGAATCACCTTGCAGATAAACTAGTACATTGGCATCGTGT	43
	LoopF	CCAGAGTTAAGATTGAT	17
	LoopB	CGAAACAAGGCCAGTTTTTTACC	23
